# Human Allogeneic Bone Marrow and Adipose Tissue Derived Mesenchymal Stromal Cells Induce CD8+ Cytotoxic T Cell Reactivity

**DOI:** 10.4172/2157-7633.S6-004

**Published:** 2013-12-12

**Authors:** Marieke Roemeling-van Rhijn, Marlies E Reinders, Marcella Franquesa, Anja U Engela, Sander S Korevaar, Helene Roelofs, Paul G Genever, Jan NM IJzermans, Michiel GH Betjes, Carla C Baan, Willem Weimar, Martin J Hoogduijn

**Affiliations:** 1 Internal Medicine, Erasmus MC, Rotterdam, The Netherlands; 2 General Surgery, Erasmus MC, Rotterdam, The Netherlands; 3 Nephrology, Leiden University Medical Center, Leiden, The Netherlands; 4 Immunohematology and bloodtransfusion, Leiden University Medical Center, Leiden, The Netherlands; 5 Department of Biology, University of York, York, United Kingdom

**Keywords:** Mesenchymal stromal cells, Bone marrow, Adipose tissue, Alloreactivity, HLA class I, CD8+ cytotoxicity

## Abstract

**Introduction:**

For clinical applications, Mesenchymal Stromal Cells (MSC) can be isolated from bone marrow and adipose tissue of autologous or allogeneic origin. Allogeneic cell usage has advantages but may harbor the risk of sensitization against foreign HLA. Therefore, we evaluated whether bone marrow and adipose tissue-derived MSC are capable of inducing HLA-specific alloreactivity.

**Methods:**

MSC were isolated from healthy human Bone Marrow (BM-MSC) and adipose tissue (ASC) donors. Peripheral Blood Mononuclear Cells (PBMC) were co-cultured with HLA-AB mismatched BM-MSC or ASC precultured with or without IFNy. After isolation via FACS sorting, the educated CD8+ T effector populations were exposed for 4 hours to Europium labeled MSC of the same HLA make up as in the co-cultures or with different HLA. Lysis of MSC was determined by spectrophotometric measurement of Europium release.

**Results:**

CD8+ T cells educated with BM-MSC were capable of HLA specific lysis of BM-MSC. The maximum lysis was 24% in an effector:target (E:T) ratio of 40:1. Exposure to IFNγ increased HLA-I expression on BM-MSC and increased lysis to 48%. Co-culturing of PBMC with IFNγ-stimulated BM-MSC further increased lysis to 76%. Surprisingly, lysis induced by ASC was significantly lower. CD8+ T cells educated with ASC induced a maximum lysis of 13% and CD8+ T cells educated with IFNγ-stimulated ASC of only 31%.

**Conclusion:**

Allogeneic BM-MSC, and to a lesser extend ASC, are capable of inducing HLA specific reactivity. These results should be taken into consideration when using allogeneic MSC for clinical therapy.

## Introduction

Mesenchymal stromal cells (MSC) are adult stemor progenitor cells which can be isolated from virtually all postnatal tissues including bone marrow and adipose tissue [1-3]. MSC are defined by their capacity to adhere to plastic, theirmultilineage differentiation capacity and a panel of cell surface markers including CD13, CD73, CD90, CD105, CD166 and HLA-class I [1,4]. HLA-class-II expression on MSC is low or absent. MSC have immune modulatory and reparative properties which makes them appealing as a cell therapeutic agent for degenerative disease and immune disorders. When exposed to inflammatory conditions such as IFNγ stimulation, MSC increase their immunosuppressive properties but also their expression of HLA-class I and II [5,6].

In the last decades, multiple clinical trials evaluating the potential of MSC for a wide spectrum of medical conditions have been conducted. Next to safety and feasibility of MSC therapy, preliminary efficacy results were obtained in some of these studies evaluating MSC amongst others in rheumatoid arthritis [7,8]; Crohn’s disease [9,10]; liver cirrhosis [11] and solid organ transplantation [12-15].

For clinical application, the choice of MSC is likely to affect the outcome of the therapy.

MSC are typically isolated from bone marrow and culture expanded. As donor age might be of influence for bone marrow derived MSC (BM-MSC) composition and function [16-18], use of MSC derived from young bone marrow donors might be preferable. Yet, this choice has some drawbacks as bone marrow aspiration is an invasive procedure and the use of BM from young individuals involves ethical dilemmas. Adipose tissue derived MSC (ASC) might serve as an alternative with several advantages: adipose tissue can be obtained in a minimal invasive manner via lipectomy or mini liposuction; adipose tissue has a higher yield of MSC [19]; and finally, ASC are at least as immunosuppressive as BM-MSC in vitro [20,21].

Next, there is a choice of using MSC of autologous or allogeneic origin. Allogeneic cells provide a practical ‘off the shelf’ cell therapeutic agent as they can be isolated and cultured in advance. Recent studies suggest that allogeneic MSC have comparable efficacy compared to autologous MSC [22,23]. However, allogeneic MSC might possibly elicit an anti-HLA immune response [24]. In contrast, autologous MSC avoid potential immunogenic responses, but need to be prepared per individual and are unsuitable for acute indications. Furthermore, although suitable in some situations [25,26], patient MSC can be affected by the disease rendering them unfit for clinical application [27,28]. In contrast, allogeneic cells provide a practical ‘off the shelf’ cell therapeutic agent as they can be isolated from healthy donors and cultured in advance. Recent studies further suggest efficacy of allogeneic MSC therapy [22,23]. However, allogeneic MSC might elicit a HLA-specific immune response [24]. The risk of anti-HLA sensitization by allogeneic MSC has been considered insignificant in the past as MSC were suggested to be low-immunogeneic [29-32]. However, we have shown that allogeneic MSC are susceptible for lysis by pre-activated CD8+ T cells [33] in an HLA-specific fashion, which challenges this old paradigm. It is however unknown whether MSC themselves are capable of inducing HLA-specific CD8+ cytotoxicity. This would signify a risk of sensitization by allogeneic MSC therapy and could potentially affect the efficacy of repeated MSC administrations or even future organs transplantations. The development of anti-MSC donor immune responses should therefore be studied before new studies are planned with allogeneic MSC. Thus, in the present study we evaluated whether repeated exposure to BM-MSC and/or ASC induced HLA-class-I specific lysis byCD8+ T cells.

## Methods

### Isolation of ASC

During live donor kidney transplantation, abdominal subcutaneous adipose tissue was surgically removed from 5 healthy kidney donors after written informed consent as approved by the medical ethical committee of the Erasmus Medical Center Rotterdam (protocol no. MEC-2006-190). Adipose tissue was collected in MEM-α medium (Sigma-Aldrich(St. Louis, MO, USA) supplemented with 100 IU/ml penicillin and 100 μg/ml streptomycin (p/s, Gibco BRL, Paisley UK) and 2 mM L-glutamine (Lonza, Verviers, Belgium). ASC were isolated as described previously (1): adipose tissue was mechanically disrupted with a scalpel knife, and then enzymatically digested with 0.5 mg/ml collagenase type IV (Sigma-Aldrich, St Louis, MO) in RPMI-1640 gluta MAX (Invitrogen) and p/s for 30 minutes at 37°C under continuous shaking. The obtained cell suspension was transferred to a T175 cm2 culture flask (Greiner Bio-one, Essen, Germany) and cultures were kept at 37°C, 5% CO2 and 95% humidity. MSC culture medium which consisted of MEM-α (Sigma-Aldrich, St. Louis, MO, USA) with 1% p/s, 2 nM L-glutamine and 15% fetal bovine serum (BioWhittaker, Verviers, Belgium) was refreshed twice a week.

When ASC cultures reached >90% confluency, MSC were detached with 0.5% trypsin-EDTA (Lonza, Verviers, Belgium) and used for experiments or frozen at −150°C until usage.

### Isolation of BM-MSC

BM was obtained from 5 hematopoietic stem cell donors after written informed consent as approved by the Medical Ethical Committee of Leiden University Medical Centre as described before [34]. In brief, BM was aspirated under general anesthesia. The mononucleated cell (MNC) fraction was isolated by Ficoll density gradient separation (Ficoll Isopaque, δ=1.077, Amersham, Uppsala, Sweden) and plated in tissue culture flasks at a density of 160 × 103 MNC/cm2 in low-glucose Dulbecco’s modified Eagle medium DMEM (Invitrogen, Paisley, UK) supplemented with 1% p/s (Lonza) and 10% fetal calf serum (FCS, Thermo Scientific HyClone,). The cultures were maintained at 37°C, 5% CO2. The medium was refreshed twice a week. When the MSC cultures became confluent, cells were collected using trypsin (Lonza) and replated at a density of 4 × 103 cells/cm2 or frozen until further usage.

### BM-MSC and ASC donor characteristics

MSC isolated from 5 healthy HLA-AB mismatched bone marrow (mean age 15.4 years, range 7-31) and adipose tissue donors (mean age 50.7 years, range 27-67) (Table 1) were used for experiments. Prior to using them in experiments, BM-MSC and ASC were cultured in parallel in MEM-with 1% p/s and 15% fetal bovine serum under standard culture conditions. When indicated, MSC were then stimulated with 100 ng/ml IFNγ for 1 week. Passage 2-6 MSC were used.

### Isolation of peripheral blood mononuclear cells (PBMC)

Peripheral blood was collected from healthy blood bank donors. Donors with 4 mismatches for HLA-A and HLA-B with the BM-MSC and ASC were selected (Table 1). PBMC were isolated by density gradient centrifugation using Ficoll Isopaque and frozen at −150°C until usage.

### Immunophenotypic characterization of BM-MSC and ASC

Unstimulated and 1 week 100 ng/ml IFNγ-stimulated BM-MSC and ASC were trypsinized and washed with FACS Flow (BD Biosciences, San Jose, CA). Cell suspensions were incubated with mouse-antihuman monoclonal antibodies against CD13-PECy7; CD45-PERCP; HLA-DR-FITC; HLA-ABC-PE-Cy7; CD31-FITC; CD73-PE; CD166-PE (all BD Biosciences); CD90-APC and CD105-FITC (R&D Systems, Abingdon, UK) at room temperature in the absence of light for 15 minutes. After two washes with FACS Flow, flow cytometric analysis was performed using 8 colors FACSCANTO-II with FACSDIVA Software (BD Biosciences) and FlowJo Software (Tree Star Inc. Palo Alto, CA). For analysis, background MFI was substracted and the mean MFI of 4 experiments were calculated.

### Generation of BM-MSC and ASC educated CD8+ effector populations

FiveBM-MSC and ASC cultures with different HLA-A and HLA-B subtypes were selected (Table 1). These BM-MSC and ASC were cultured in parallel under the same conditions for 1 week with or without 100 ng/ml IFNγ before they were seeded in 24-well flat bottom plates. PBMC with a 2-2 mismatch for HLA-A and HLA-B with both the BM-MSC and the ASC were then selected. To generate effector cells, 5 × 105 PBMC were co-cultured with 1,2 × 106 BM-MSC or, in parallel, with the ASC in MEMα containing 10% of human heat inactivated serum and 200 IU/mL IL-2 (Chiron, Amsterdam, The Netherlands). After 1 week of co-culture, the PBMC were removed from the co-cultures, washed with 1× PBS and incubated with mononuclear antibodies against CD3-Amcyan, CD8-PE-Cy7 and 7AAD-viaprobe (all BD Biosciences) for 15 minutes in the dark. The cells were washed and the BM-MSC and ASC educated effector CD3+CD8+ cell populations were isolated by FACS sorting (FACS-ARIA Cell-sorter, BD Biosciences).

**Figure 1:**
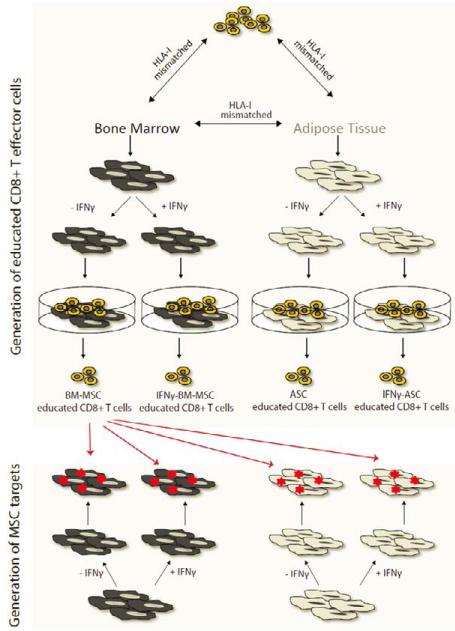
Experimental design of study. Five HLA-class I mismatched BM-MSC and ASC pairs were used. BM-MSC and ASC were either unstimulated or pre-stimulated for 1 week with IFNγ. To obtain educated CD8+ T cells, a co-culture was established with BM-MSC or ASC and HLA-class I mismatched PBMC in the presence of 200 U/ml IL-2. CD3+CD8+ effectors (BM-MSC educated CD8+ T cells; IFNγ-BM-MSC educated CD8+ T cells; ASC educated CD8+ T cells and IFNγ-ASC educated CD8+ T cells) were selected via FACS sorting. CD3+CD8− cells were isolated from all co-cultures as a negative control. After Europium labeling, target cells, either IFNγ-stimulated or unstimulated BM-MSC and ASC, were exposed to the effector cells as depicted here for BM-MSC educated CD8+ T cells. Europium release was assessed as a measure of CD8+ T cell mediated lysis.

This procedure resulted in the isolation of the following effector populations: CD3+CD8+ cells educated by BM-MSC (BM-MSC educated CD8+ T cells); CD3+CD8+ cells educated by IFNγ-stimulated BM-MSC (IFNγ-BM-MSC educated CD8+ T cells); CD3+CD8+ cells educated by ASC (ASC educated CD8+ T cells) and CD3+CD8+ cells educated by IFNγ-stimulated ASC (IFNγ-ASC educated CD8+ T cells); as a control, the CD3+CD8− populations were isolated from all four co-cultures. Our experimental design is depicted in Figure 1.

### Evaluation of cytotoxicity mediated lysis of BM-MSC and ASC by Europium release assay

To examine the cytotoxic capacity of the CD3+CD8+ effector T cells, BM-MSC and ASC identical to those used in the co-cultures were cultured for 1 week in the presence or absence of 100 ng/ml IFNγ andlabeled with Europium-diethylenetriaminepentaacetate (DTPA) (Sigma-Aldrich, St. Louis, MO) (Figure 1). These target MSC were used in the Europium release cytotoxicity assay as described previously [33]. In brief, the different effector populations were exposed for 4 hours to each of the different Europium labeled targets cells; unstimulated BM-MSC; IFNγ-stimulated BM-MSC; unstimulated ASC and IFNγ-stimulated ASC. The effectors were incubated with 2500 target cells at effector:target (E:T) ratios of 40:1 to 0.3:1 in round-bottom 96-well plates (Nunc, Roskilde, Denmark) at 37°C. The plates were then centrifuged and 20 μl of the supernatant was transferred to 96-well plates with low background fluorescence (fluoroimmunoplates [FluoroNunc plates]; Nunc). Subsequently, 100 μl of enhancement solution (PerkinElmer, Groningen, The Netherlands) was added to each well and release of Europium was measured in a time-resolved fluorometer (Victor 1420 multilabel counter; LKB-Wallac, Turku, Finland). Maximal release of Europium by target cells was measured by incubation of 2500 labeled target cells with 1% Triton (Sigma-Aldrich, Zwijndrecht, the Netherlands) for 4 hr. Spontaneous release of Europium was measured by incubation of labeled target cells without effector cells for 4 hr; the percentage leakage was calculated as (spontaneous release/maximal release) × 100%. The percentage cytotoxicity mediated lysis was calculated as %lysis=(measured lysis – spontaneous release)/(maximal release – spontaneous release) × 100%.

### Statistical analysis

Paired t-test was used to test for statistical significance in MFI of the staining for cell surface markers on BM-MSC and ASC. Two-way ANOVA was used to evaluate statistical significance of differences in lysis of different target populations and by the CD8+ T effector cells and CD8− T cell control populations. Significant lysis was defined as lysis of a target cell by a certain CD8+ T effector population which was significantly higher than the background lysis of the CD8− negative control T cell population.

## Results

### BM-MSC and ASC immunophenotype

Cell surface marker expression was analyzed by flow cytometryusing a characterization panel for MSC. Both BM-MSC and ASC expressed CD13; CD73; CD90; CD105; CD166 and HLA-ABC and were negative for HLA-DR; CD31 and CD45, confirming their MSC immunophenotype (Figure 2A).

Expression of HLA-class I was upregulated in BM-MSC and ASC after IFNγ stimulation (Figure 2B). IFNγ stimulation did not affect MSC cell surface marker expression on ASC. In BM-MSC, only CD13 was slightly upregulated after IFNγ stimulation (data not shown).

### BM-MSC induce HLA-class I specific lysis by CD8+ T cells

**Figure 3ABC:**
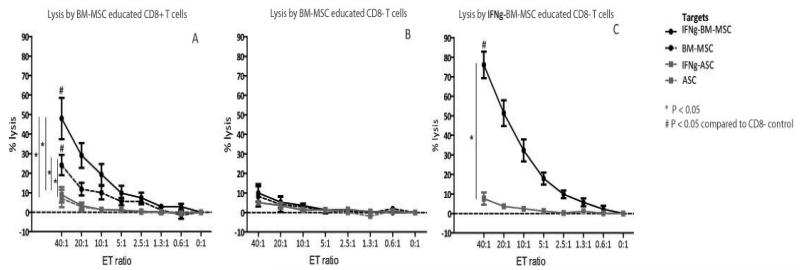
**A**: Dose dependent lysis of BM-MSC by BM-MSC educated CD8+ T cells (black dashed line). Lysis increased when BM-MSC targets were IFNγ-stimulated (black solid lines) and there was no lysis of the mismatched ASC (grey lines) indicating HLA-class I specific lysis. Mean ± SEM, n=5. **B**: The BM-MSC educated CD8− T cells were not capable of lysing any of the target populations. Mean ± SEM, n=5. **C**: When CD8+ T cells were educated with IFNγ-stimulated BM-MSC, lysis of IFNγ-stimulated BM-MSC targets increased (black solid line). Lysis was HLA-class I specific as IFNγ-BM-MSC educated CD8+ T cellsdid not lyse the mismatched ASC (grey lines). Mean ± SEM, n=5.

To evaluate the capacity of BM-MSC to induce CD8+ T cell mediated lysis, CD8+ T effector cells were isolated via FACS sorting after one week of co-culture of PBMC with HLA-AB mismatched BM-MSC. BM-MSC educated CD8+ T effector cells were capable of lysing Europium labeled BM-MSC identical to the one used in the co-culture (Figure 3A). Lysis was dose dependent and reached a maximum of 24% (mean, range 13-37%) at a 40:1 effector:target (E:T) ratio, which was significantly higher than lysis byCD8− T cells (Figure 3B). IFNγ stimulation of BM-MSC targets further increased lysis to 48% (mean, range 25-80%, Figure 3A). In contrast, when BM-MSC-educated CD8+ T cells were exposed to IFNγ-stimulated or unstimulated ASC with a different HLA make-up, no lysis was detected (Figure 3A) indicating that the BM-MSC induced lysis was HLA-class I specific.

### IFNγ stimulation increases BM-MSC inducedlysis

CD8+ T effector cells were educated with IFNγ-stimulated BM-MSC to study the effect of inflammatory conditions on the induction of CD8+ T cell mediated lysis. IFNγ-BM-MSC educated CD8+ T cells induced 76% lysis of IFNγ-stimulated target BM-MSC (mean, range 58-91%) (Figure 3C), which was significantly higher than the lysis induced by unstimulated BM-MSC (Figure 3A). No lysis of ASC with different HLA was observed (Figure 3C) confirming the HLA-class I specific character of the lysis.

### ASC induce less lysis compared to BM-MSC

**Figure 4ABC:**
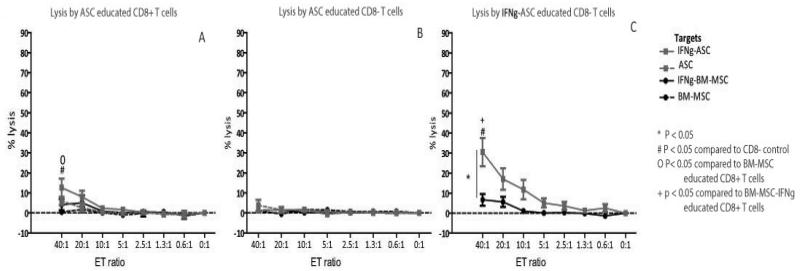
**A**: CD8+ T cells educated with ASC were only capable of lysis of IFNγ-stimulated ASC (grey solid line) and not of the unstimulated ASC (grey dashed lines) or the mismatched BM-MSC (black lines). Mean ± SEM, n=5. **B**: The ASC educated CD8− T cells did not lyse any of the target populations. Mean ± SEM, n=5. **C**: IFNγ-ASC educated CD8+ T cells were capable of significant and dose dependent lysis of IFNγ-stimulated ASC (grey solid line) and not of the mismatched BM-MSC (black line). This lysis was markedly lower than the lysis induced by IFNγ-stimulated BM-MSC (figure 3C, black solid line). Mean ± SEM, n=5.

Next, we studied the capacity of ASC to induce CD8+ T cell mediated lysis. Educated CD8+ T effector cells were generated by co-culturing ASC and mismatched PBMC. In contrast to BM-MSC educated CD8+ T cells, which significantly lysed BM-MSC, ASC-educated CD8+ T cells were only capable of lysing ASC when target ASC were pre-stimulated with IFNγ (mean 13%, range 4-21, Figure 4A).This lysis of IFNγ-stimulated ASC target cells was significantly lower than the lysis of IFNγ-stimulated BM-MSC targets (Figure 3A by BM-MSC-educated CD8+ T cells (Figure 3A).

### IFNγ stimulation increases ASC induced lysis

Finally, to evaluate the influence of inflammatory conditions on ASC on their capacity to induce CD8+ cytotoxicity, PBMC were co-cultured with IFNγ-stimulated mismatched ASC. The IFNγ-ASC educated CD8+ T cells were capable of significant lysis of IFNγ-stimulated ASC targets (mean lysis of 31%, range 19-51%, Figure 4C) and not of the mismatched IFNγ-stimulated BM-MSC (Figure 4C). These data confirm the HLA-class I specificity of ASC induced lysis and further denote an effect of IFNγ stimulation on the ASC induced CD8+ mediated lysis, as also seen in the experiments performed with BM-MSC. However, the maximum lysis of 31% IFNγ stimulated ASC by IFNγ-ASC educated CD8+ T cells was substantially lower than the maximum lysis of 76% of IFNγ-stimulated BM-MSC by IFNγ-BM-MSC educated CD8+ T cells. Therefore, these results indicate a lower capacity of IFNγ-stimulated ASC to induce CD8+ cytotoxity compared to IFNγ-stimulated BM-MSC.

## Discussion

Currently, MSC have been applied in several clinical studies. Some of these studies indicated a clinical effect of MSC while others could not confirm MSC efficacy [12,35-37]. These contradicting outcomes might be explained by a great variety in MSC preparations. MSC of bone marrow or adipose tissue and of autologous as well as allogeneic origin have been used. The effect of these differences in MSC preparation on study outcome is currently unknown. To decide on the use of allogeneic or autologous MSC, more knowledge on immunogenicity of allogeneic MSC is required and potential differences between bone marrow and adipose tissue derived MSC need to be evaluated.

The use of allogeneic MSC has several benefits. It can be used as an ‘off the shelf’ therapy and recent studies suggest that allogeneic MSC can provide an effective treatment [22,23]. And, as several preclinical studies found MSC to be low immunogeneic [29-32], it has been suggested that allogeneic MSC can be used without risk for sensitization. However, other studies showed that allogeneic MSC are susceptible for lysis by CD8+ T cells [38] and NK cells [38,39] and can induce memory T cells [40-42] and the production of IgG antibodies [43], which argues against a low-immunogeneic profile of MSC. In the current study we investigated whether MSC are capable of inducing HLA-class I specific CD8+ T cell cytotoxicity. We tested this for the most commonly used bone marrow derived MSC as well as adipose derived MSC, which are an important alternative.

In this study, we generated educated CD8+ T cells by co-culturing BM-MSC with HLA-AB mismatched PBMC, simulating the first exposure of allogeneic MSC to the recipient immune system. Next, we challenged those educated CD8+ effector T cells with MSC from the same donor that were used in the co-culture, to mimic a second exposure to MSC, or with MSC from an allogeneic HLA-AB mismatched donor. In this system, we found BM-MSC capable of inducing of HLA-class I specific lysis of allogeneic BM-MSC. This indicates that though allogeneic BM-MSC can be immunosuppressive in vitro [29], activation of the adaptive immune system by BM-MSC is not prevented by this anti-proliferative effect.

When used for treatment of inflammatory disease, MSC might face an inflammatory environment. Inflammatory conditions, which can be simulated with IFNγ, increase the immunosuppressive potential of MSC [6,44]. However, in accordance with others [5], we found that BM-MSC and ASC also increase their expression of HLA-class I upon IFNγ stimulation. This upregulation could influence the induction of CD8 cytotoxicity as this is a HLA-class I restricted process. Thus, we used CD8+ T cells educated with IFNγ-stimulated BM-MSC and found that the lysis of IFNγ-stimulated BM-MSC was increased. This implies that inflammatory conditions in vivo might increase the immunogenicity of BM-MSC.

Next, we evaluated the immunogenicity of ASC. ASC are arising as an alternative for BM-MSC as they have several favorable characteristics: adipose tissue is easy to obtain and has a high yield of MSC; further, ASC are at least as immunosuppressive in vitro as BM-MSC [20,21]. Surprisingly, we found that ASC were not capable of inducing CD8+ T cell mediated lysis. Significant lysis was only detected when ASC targets were IFNγ-stimulated. This indicates a less immunogeneic profile of ASC compared to BM-MSC as BM-MSC did induce significant CD8+ T cell mediated lysis. When using IFNγ-stimulated ASC to educate the CD8+ T cells, an increase in lysis of ASC was observed. However, this lysis was again markedly lower than the lysis induced by IFNγ-stimulated BM-MSC, confirming the less immunogeneic profile of ASC. We hypothesized that this difference in immunogenicity could be the result of levels of HLA-class I expression. Yet, although BM-MSC showed a higher fold increase in HLA-class I expression upon IFNγ stimulation, HLA-class I levels were lower in unstimulated BM-MSC compared to ASC. Another potential explanation for the difference in lysis induced by BM-MSC and ASC might be found in the differences in age of BM-MSC and ASC donors used in this study. Age has been suggested to be of relevance for the differentiation capacity and composition of BM-MSC [16,17]. However the proliferation rate of ASC was found to be unaffected by age [45] and the effect of age on other properties of BM-MSC and ASC such as the immunosuppressive capacity as well as their immunogeneic potential remains to be determined. Finally, the difference in immunogenicity between BM-MSC and ASC can be explained by for instance a difference in the expression of lysis inhibiting proteins such as serpins remains to be determined.

Taken together, our results indicate that BM-MSC and ASC can induce anti HLA sensitization. Whether this property will translate into sensitization when MSC are infused in vivo is unknown. It can be questioned whether MSC reside long enough after infusion to initiate this immune response [46]. Yet, the potential of MSC to induce anti HLA sensitization as described in this study plea for the use of autologous MSC treatment if possible and a careful consideration when applying allogeneic MSC. In particular situations, the use of allogeneic MSC is preferable such as in situations of acute organ failure or in cases when the patients’ disease affect MSC functionality. In those situations, the risk of allogeneic MSC therapy can possibly be reduced by choosing a low risk study design e.g. using allogeneic MSC which are mismatched with the organ donor and screening of MSC recipients for absence of anti-donor reactivity prior to MSC treatment and monitoring for the development of anti-MSC donor immune responses after MSC treatment.

## Figures and Tables

**Figure 2A:**
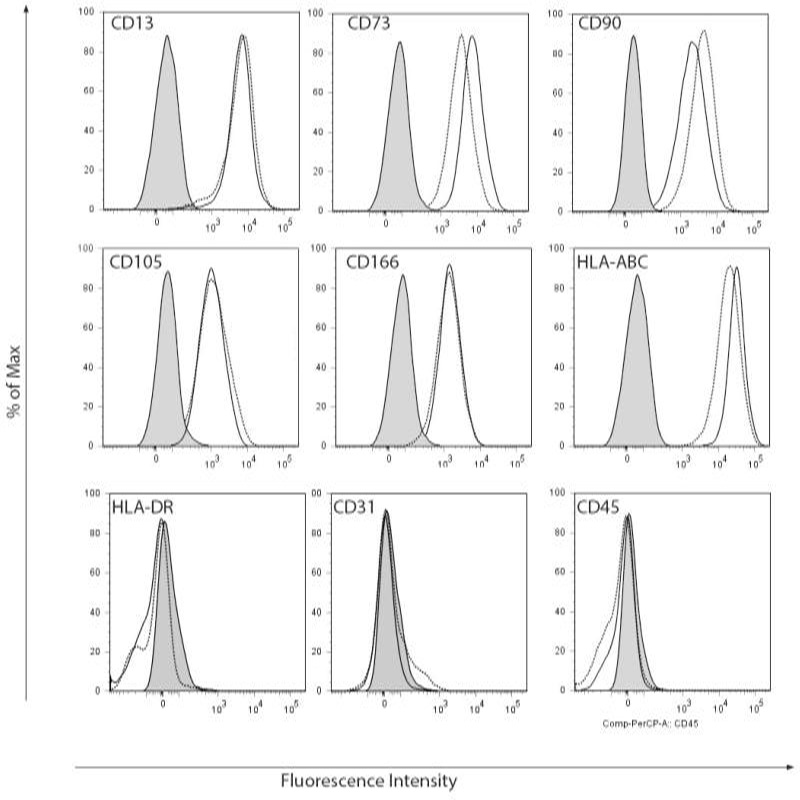
Flowcytometric immunophenotyping of BM-MSC and ASC. BM-MSC (black solid lines) and ASC (black dotted lines) expressed MSC markers CD13, CD73, CD90, CD105, CD166 and HLA-ABC and were negative for HLA-DR, CD31 and CD45. No significant differences were detected. Grey solid histograms represent unstained control, n=4, representative examplesare shown.

**Figure 2B:**
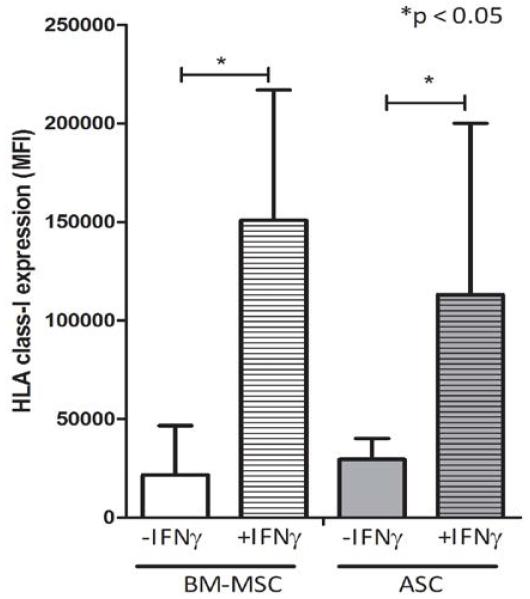
IFNγ stimulation resulted in upregulation of HLA-class I expression on BM-MSC and ASC, mean MFI of n=5, *p<0.05.

**Table 1:** HLA typing of BM-MSC, ASC and PBMC used in the 5 experiments.

No	BM-MSC/ASC	HLA-I		HLA-I	
		A	A	B	B
1	BM-MSC 1	1	3	7	35
	ASC 1	24		15	51
	PBMC 1	32(19)		44(12)	18
2	BM-MSC 2	3	24(9)	35	38(16)
	ASC 2	1	30(19)	8	41
	PBMC 2	68(28)		51 (5)	53
3	BM-MSC 3	24(9)		62(15)	55(22)
	ASC 3	11	30(19)	52(5)	35
	PBMC 3	1	3	8	64(40)
4	BM-MSC 4	2	29(19)	7	44(12)
	ASC 4	1	26(10)	38(16)	51(5)
	PBMC 4	3	23	49	61
5	BM-MSC 5	2	24(9)	35	58(17)
	ASC 5	3		7	13
	PBMC 2	68(28)		51 (5)	53
